# Co expression of SCF and KIT in gastrointestinal stromal tumours (GISTs) suggests an autocrine/paracrine mechanism

**DOI:** 10.1038/sj.bjc.6603063

**Published:** 2006-03-28

**Authors:** N Théou-Anton, S Tabone, D Brouty-Boyé, R Saffroy, L Ronnstrand, A Lemoine, J-F Emile

**Affiliations:** 1 INSERM U602, Villejuif, France; 2 INSERM U602, INSERM U590, Centre Léon Bérard, Lyon, France; 3 INSERM U602, Villejuif, France; 4 AP-HP, Hôpital Paul Brousse, Biochemistry and Molecular Biology Department, INSERM U602, Villejuif, France; 5 Lund University, Experimental Clinical Chemistry, Department of Laboratory Medicine, Malmö University Hospital, Malmö, Sweden; 6 Biochemistry and Molecular Biology Department, AP-HP, Hôpital Paul Brousse, INSERM U602, Villejuif, France; 7 Pathology Department, AP-HP, Hôpital Ambroise Paré, UVSQ, Faculté de Médicine PIFO, INSERM U602, Boulogne 92104, France

**Keywords:** gastrointestinal tumour, sarcoma, SCF, autocrine loop

## Abstract

KIT is a tyrosine kinase receptor expressed by several tumours, which has for specific ligand the stem cell factor (SCF). *KIT* is the main oncogene in gastrointestinal stromal tumours (GISTs), and gain-of-function *KIT* mutations are present in 70% of these tumours. The aim of the study was to measure and investigate the mechanisms of KIT activation in 80 KIT-positive GIST patients. KIT activation was quantified by detecting phosphotyrosine residues in Western blotting. SCF production was determined by reverse transcriptase–PCR, ELISA and/or immunohistochemistry. Primary cultures established from three GISTs were also analysed. The results show that KIT activation was detected in all cases, even in absence of *KIT* mutations. The fraction of activated KIT was not correlated with the mutational status of GISTs. Membrane and soluble isoforms of SCF mRNA were present in all GISTs analysed. Additionally, SCF was also detected in up to 93% of GISTs, and seen to be present within GIST cells. Likewise, the two SCF mRNA isoforms were found to be expressed in GIST-derived primary cultures. Thus, KIT activation in GISTs may in part result from the presence of SCF within the tumours.

Proto-oncogene KIT is a class III transmembrane tyrosine kinase receptor (TKR) (Reilly, 2003) which is involved in the growth and differentiation of haematopoietic stem cells, mast cells, melanocytes, germinal cells and Cajal's interstitial cells (Ashman, 1999). The binding of KIT ligand promotes KIT dimerization, and activates its intrinsic tyrosine kinase activity, thereby resulting in a transphosphorylation at several critical tyrosine residues and an activation of downstream signal transduction molecules (Blume-Jensen *et al*, 1991). The ligand for KIT receptor is the stem cell factor (SCF) also known as KIT ligand, steel factor and mast cell growth factor. SCF is implicated in cell proliferation (Williams and Allan, 1996; Drayer *et al*, 2005), migration and survival (Bredin *et al*, 2003; Erlandsson *et al*, 2004). Recently, SCF has also been shown to be implicated in a resistance mechanism of malignant mesothelioma cells to drug treatment (Catalano *et al*, 2004). SCF expression is widely distributed throughout the body, especially in stromal cells such as fibroblasts and endothelial cells, and detectable at low levels in the blood (Ashman, 1999). SCF exists under soluble (s) and membrane-bound (m) forms due to differential splicing and proteolytic cleavage (Broudy, 1997). The two forms display distinct effects as regards to the survival and proliferation of haematopoietic cell lines (Caruana *et al*, 1993; Miyazawa *et al*, 1995) and primary cells (Fujita *et al*, 1989; Toksoz *et al*, 1992), although they are both active in increasing the number of human progenitor cells in the context of stromal cell cultures (Toksoz *et al*, 1992). Stromal mSCF appears to induce more persistent signalling than the soluble form, this last form inducing rapid downregulation of cell surface expression and degradation of KIT (Miyazawa *et al*, 1995).

KIT is detectable in several human tumours (Went *et al*, 2004), and paracrine/autocrine activation by its ligand has been suggested to be involved in numerous malignancies, including small-cell lung cancer (Hibi *et al*, 1991; Krystal *et al*, 1996), ovarian cancer (Inoue *et al*, 1994), neuroblastoma (Ricotti *et al*, 1998), breast carcinoma (Hines *et al*, 1995), leukaemia (Pietsch, 1993; Zheng *et al*, 2004), colon carcinoma (Lahm *et al*, 1995) and Leydig cell tumour (Kondoh *et al*, 1995). Gastrointestinal stromal tumours (GISTs) are the most frequent mesenchymal tumours of the digestive tract. Nearly all of them express KIT and 70% have a gain-of-function mutation of *KIT* gene (Corless *et al*, 2004), responsible for ligand-independent KIT activation (Hirota *et al*, 1998). Moreover, rare GISTs have been shown to be related to familial *KIT* mutations (Nishida *et al*, 1998), and transgenic mice with a gain-of-function mutation of *KIT* have a high incidence of GIST (Sommer *et al*, 2003). Finally, treatment of GIST patients with the KIT inhibitor Imatinib can induce tumour regression in many patients (Heinrich *et al*, 2003a). Thus, KIT activation plays a major role in GIST oncogenesis. Surprisingly, there are a few data dealing with SCF in GISTs and its possible role as an autocrine/paracrine growth factor.

In the present study, we quantified the activation of KIT in GIST samples and found that there is no correlation with the presence of gain-of-function mutations. We also show that the two isoforms of the KIT ligand SCF are present in nearly all GISTs, and are produced by tumour cells.

## MATERIALS AND METHODS

### Patients

A total of 80 patients with GIST were included in the study. For all cases, GIST diagnosis was confirmed by at least two pathologists. Haematoxyline–eosin–saffran staining and immunohistochemistry were performed with CD117/KIT (rabbit polyclonal, Dako, Copenhagen, Denmark, 1 : 300 dilution), CD34 (mouse, clone QBEND, Immunotech, Marseille, France, 1 : 2 dilution), and S100 protein (rabbit polyclonal, Dako, 1 : 200 dilution). Detection of exon 9, 11, 13, 17 mutations of *KIT*, and exon 12 and 18 mutations of *PDGFRA* was performed on either formalin fixed or frozen tumour samples as previously described (Emile *et al*, 2002, 2004). Clinical and pathological characteristics, as well as mutational status of most cases, have been previously reported (Emile *et al*, 2004; Théou *et al*, 2004; Tabone *et al*, 2005). Frozen and fresh tumour samples were obtained from 18 and three patients, respectively. Tumour samples were collected during surgical resection performed for therapeutic purposes and harvested before treatment with imatinib mesylate. According to French ethical laws, none of the patients expressed his willingness not to be included in this study. Paraffin-embedded and frozen normal digestive tissue samples from eleven patients without GIST were used as controls. None of them displayed mutations in *KIT* or *PDGFRA*.

### Cell isolation and primary cell culture

Fresh GIST samples were minced with scissors, washed twice in PBS, and incubated in a 0.25% solution of collagenase A (Roche Diagnostics, Meylan, France) at +37°C until disaggregation was complete. After cold centrifugation in PBS, cell pellets were resuspended in 1640 RPMI medium supplemented with 10%s FCS (both from Gibco BRL, Cergy-Pontoise, France), and seeded into culture flasks (ATGC Biotechnologie, Noisy-le-Grand, France) at approximately 1.0 × 10^5^ cells cm^−2^. Medium changes were performed 24 h after seeding, and once a week before cell analysis. For RNA extraction, confluent cultures were trypsinized, and cells were centrifuged at 2000 r.p.m. for 5 min at room temperature. Cell pellets were vortexed in lysis buffer (350 *μ*l/10^6^ cells) of RNeasy KIT for RNA extraction (Qiagen, Courtaboeuf, France), and kept at −80°C before analysis.

### Detection of soluble and membrane-bound forms of SCF mRNA

For RNA extraction, frozen tissues were mechanically homogenized (Mixer Mill MM 300, Retsch, Germany). RNA was extracted from homogenates of tumours, digestive tissues and cultured cells with the RNeasy & QIAshredder KITs (Qiagen) according to the manufacturer's instructions. A maximum of 1 *μ*g of RNA was reverse transcribed in a 20 *μ*l final volume. The reaction mixture contained 1 × RT buffer, 5.5 mM MgCl_2_, 500 *μ*M each dNTP, 2.5 *μ*M random hexamers, 0.4 U *μ*l^−1^ RNase inhibitor and 1.25 U *μ*l^−1^ reverse transcriptase (Applied Biosystems, Foster City, CA, USA). The cycling conditions were 10 min at 25°C, 30 min at 48°C, and 5 min at 95°C. Soluble and membrane-bound SCF mRNA were detected by PCR amplification using primers SCF-F-HEX (5′CAAGGACTTTGTAGTGGCATCT3′) and SCF-R (5′GAGAAAACAATGCTGGCAAT3′) on RT–PCR products. Each of the 35 PCR cycles consisted of denaturation for 1 min at 92°C, annealing for 30 s at 60°C and elongation for 45 s at 72°C. Each PCR included an initial denaturation step of 2 min at 94°C and a final elongation step of 7 min at 72°C. To detect the PCR HEX-labelled products and to determine their size, 1 *μ*l of each sample was added to 23 *μ*l of formamide and 0.5 *μ*l of GeneScan 500 TAMRA size standard (Applied Biosystems, Foster City, CA, USA). Capillary electrophoresis was performed with an ABI Prism 310 apparatus, according to the manufacturer's instructions. Size curves and fluorescence intensity were analysed and quantified with Genescan software (Applied Biosystems).

### Immunohistochemical detection of SCF

Paraffin-embedded donor tissue blocks were sampled with 0.6 punchers using a tissue microarray (TMA) instrument (Beecher Instruments Inc., Sun Prairie, WI, USA). Previously haematoxylin–eosin–saffran stained sections were used to select representative tumour areas, and for each tumour, three cylindrical cores were removed from the donor block and placed into the recipient TMA paraffin block. After antigen removal, TMA sections were subjected to immunohistochemical staining by using anti-KIT (1 : 300 dilution), DOG-1 (rabbit polyclonal S0284, Applied Genomics Inc., Huntsville, AL, USA, 1 : 40 dilution) and SCF (rabbit polyclonal H-189, Santa Cruz Biotechnology Inc., Santa Cruz, CA, USA, 1 : 40 dilution) antibodies, and avidin–biotin complex immunoperoxidase technique (LSAB2, Dako). Immunohistochemistry was also performed on 10 large GIST samples, which contained tumour as well as adjacent nontumour tissue.

### Protein extraction and analysis

Frozen GIST samples were calibrated and mechanically homogenized (Mixer Mill MM 300) in lysis buffer (20 mM Tris, 150 mM NaCl, 1 mM othovanadate, 10 mM NaF, 1 mM PMSF, 0.5 *μ*g ml^−1^ leupeptine, 1 *μ*g ml^−1^ pepstatine, 10 KIU ml^−1^ aprotinine, 1% Triton X-100). Lysates were rocked for 30 min at 4°C, and then centrifuged to remove insoluble material. Supernatant protein contents were determined using Bradford solution and were normalized.

KIT was immunoprecipitated from 100 *μ*l of lysates, using anti-CD117 antibody and Sepharose protein G beads (Amersham Pharmacia Biotech, Les Ulis, France). After 1 h incubation at 4°C under constant agitation, the beads were washed three times in lysis buffer. Immunoprecipitates and total lysates were resuspended in Laemmli buffer, heated and separated by 5–15% SDS–polyacrylamide gel electrophoresis under reducing conditions, and transferred to a polyvinylidene difluoride membrane (Biorad, Marnes-la Coquette, France). Western blots on immuno-precipitates were performed using 4G10 (kindly provided by Dr C Boucheix, 1 : 5 dilution) phosphotyrosine mAb and anti-CD117 (rabbit polyclonal, Dako, 1 : 1000 dilution) antibody whereas Western blots on total lysates was performed using TYR 721 Antibody (Voytyuk *et al*, 2003, rabbit polyclonal, 1 *μ*g ml^−1^)). Immunoreactive bands were visualized by using appropriate secondary horseradish peroxidase-conjugated antibodies (Immunotech, Marseille, France) and enhanced chemiluminescence (PerkinElmer, Life Sciences). Measurement of fluorescent intensity was performed by using Intelligent Dark box II (Fujifilm, FUJI, Japan) and Image Gauge V4.0 5F (Fujifilm) sofware. Quantitative determinations of human SCF concentrations were performed using 100 *μ*l of protein extracts by ELISA (Quantikine, R&D systems, USA) according to the manufacturer's instructions.

## RESULTS

### KIT activation in GISTs

**Figure 1:**
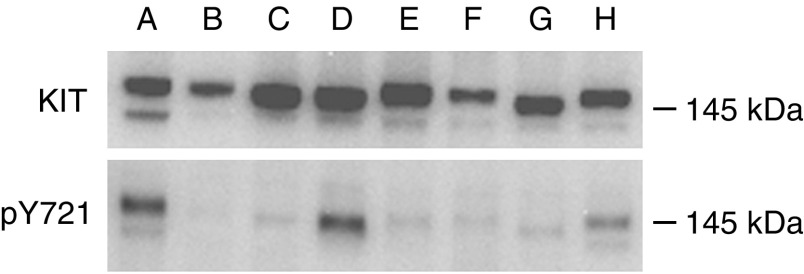
Expression of phosphorylated Tyrosine 721 of *KIT* protein. For each sample, 18 *μ*g of total protein extracts were loaded in gels and revealed on Western blot by Y721 polyclonal antibody specific for the phosphorylated tyrosine 721 (upper gel) or polyclonal anti-KIT antibody (lower gel). Wild type GIST: A (71105) and B (71183). Mutated GIST C (71229), D (71175), E (71100), F (71237), G (70810) and H (71101).

**Figure 2:**
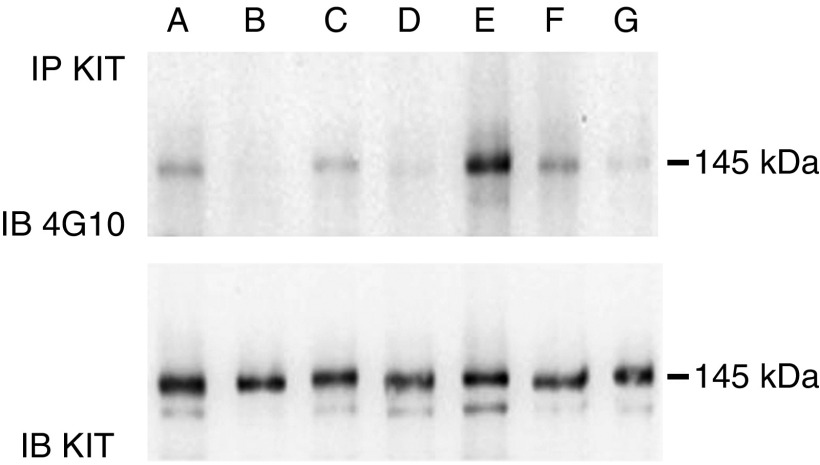
Expression of phosphorylated *KIT* protein. Protein extracts were immunoprecipitated by KIT antibody. A first Western blot was revealed with KIT antibody to quantify the amount of KIT in each samples. Appropriate dilutions were then performed to load the same amount of KIT from each samples in a second gel revealed by 4G10 mAb (upper gel) and a third gel revealed by KIT (lower gel). On the upper gel, the bands of 145 kDa corresponded to activated KIT protein. The lower gel confirmed that similar amounts of total KIT were loaded from each samples. Wild-type GIST: A (71186) and B (71105). Mutated GIST C (71237), D (71101), E (70810), F (71231) and G (71562).

KIT activation was determined in total protein extracts prepared from 17 frozen GIST samples by two different methods. In the first step, analyses was performed by Western blotting using TYR721 antibody, specific for the phosphorylated tyrosine 721. A positive signal was detected in 14/17 cases (Figure 1). However, the total amount of activated KIT highly varied between tumours. To quantify the fraction of activated KIT present in each GIST sample, total protein extracts were immunoprecipitated with anti-KIT antibody. After Western blotting, dilutions were made to obtain the same amount of KIT in each sample. KIT activation was then analysed on a second Western blot using 4G10 mAb, specific for phosphotyrosine. As illustrated in Figure 2, a positive signal was detected at 145 kDa in all cases and the fraction of KIT activation differed between samples. The fraction of activated KIT revealed by 4G10 mAb was then quantified in GIST samples (Table 1), no correlation was found with the presence of *KIT* mutations (*P*>0.05, nonparametric Wilcoxon test).

### Detection of SCF in GISTs

As KIT activation did not depend on the gain-of-function mutations, we looked for the presence of the KIT ligand SCF within the tumours. Indeed, SCF mRNAs were detected in the 18 frozen GISTs samples tested by RT–PCR. SCF was also detected in protein extracts from eight GISTs by ELISA with concentrations ranging from 3.7 to 89 pg ml^−1^ of total protein extract (mean 29.6 pg ml^−1^). Interestingly, the lowest SCF amount was found in a tumour in which *PDGFRA* mutation had been detected. To further confirm the presence of SCF in GISTs, we performed immunohistochemistry on tissue microarray containing 67 GISTs positive for both KIT and DOG-1. In all, 62 cases (93%) were positive for SCF.

**Figure 3:**
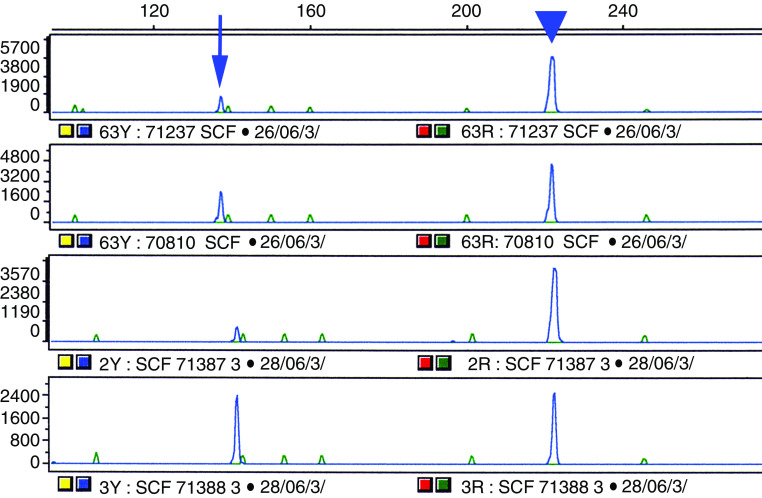
Quantification of SCF isoforms by fluorescence intensity after RT–PCR in GISTs and digestive tissues. Fluorescent amplicons (blue peaks) were separated by capillary electrophoresis and identified on the basis of their size. Peaks at 222 base pairs (arrow head) corresponded to sSCF, while peaks at 137 base pairs (arrow) corresponded to mSCF. The green peaks corresponded to size markers (TAMRA). Numbers 71237 and 70810 are GISTs samples, and 71387 and 71388 are digestive tissues from muscularis propria (ileon) and mucosa (colon), respectively.

As seen in Figure 3, primers for RT-PCR allowed amplifying the two SCF isoforms. The larger amplicon (222 base pairs) corresponded to the sSCF, containing a proteolytic domain. The smaller amplicon (137 base pair) did no contain this domain and corresponded to the mSCF. sSCF signal was higher in most (14/18) cases.

### Production of SCF by tumour and nontumour cells

SCF is known to be produced by several types of cells. Indeed, SCF mRNA was detected in the 11 normal digestive control samples that we tested (Table 1). SCF was also detected by ELISA in two of three control digestive samples.

**Figure 4:**
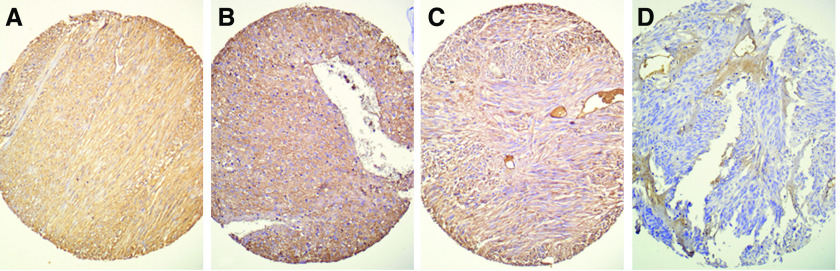
Detection of SCF staining within GIST cells by immunohistochemistry with anti-SCF antibody. A positive diffuse staining was present in most GISTs (**A–C**), but absent in some cases (**D**). The staining was generally both cytoplasmic and membranous (**A**), and sometimes predominantly membranous (**B**) or cytoplasmic (**C**).

Immunohistochemistry analysis showed that, in the 63 GIST samples positive for SCF, the staining was mainly detected within tumour cells. In nearly all positive cases, cellular staining was diffuse, and generally displayed a membranous and cytoplasmic distribution pattern (Figure 4).

**Figure 5:**
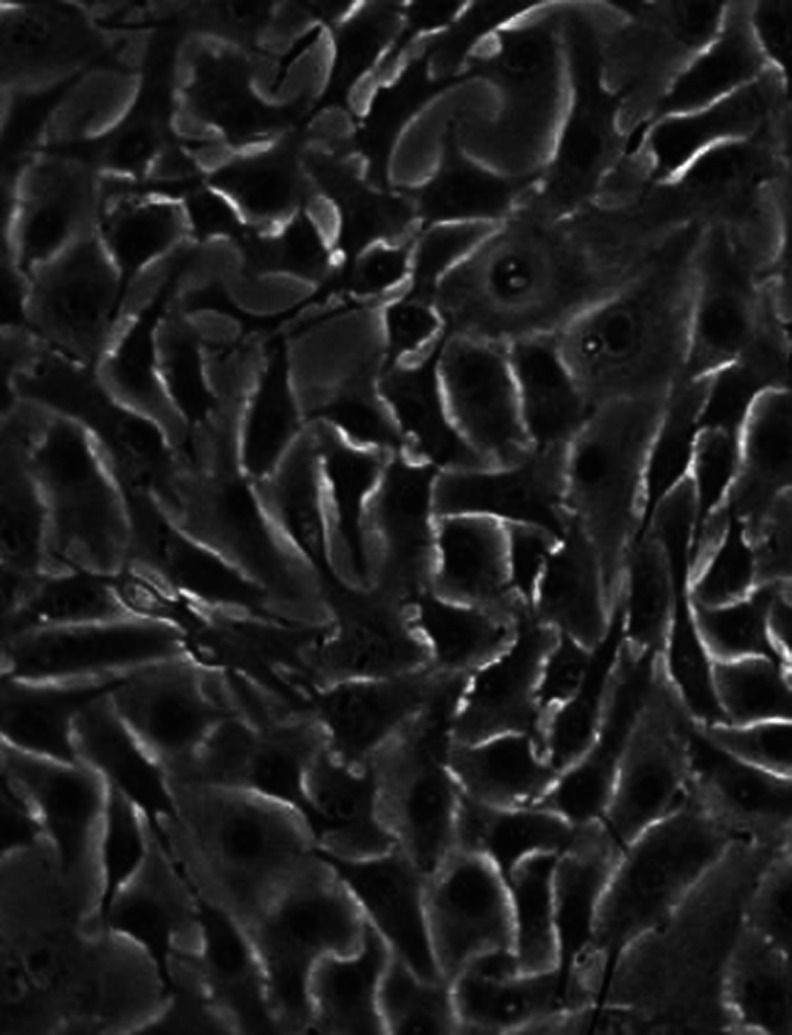
Primary cultures of GIST cells. Primary cultures of GISTs showed a homogeneous population of spindle-shaped cells (primary culture of GIST 70810, original magnification × 400).

To further confirm that GIST cells were able to produce SCF, primary cultures were established from three fresh tumours derived from the stomach, and all strongly positive for KIT. Primary cultures consisted of a homogeneous population of spindle-shaped cells (Figure 5). The heterozygous *KIT* exon 11 deletions detected in two out of the three tumours, were similarly present in the corresponding primary cultures. Moreover, RT–PCR also revealed the presence of both both sSCF and mSCF.

**Figure 6:**
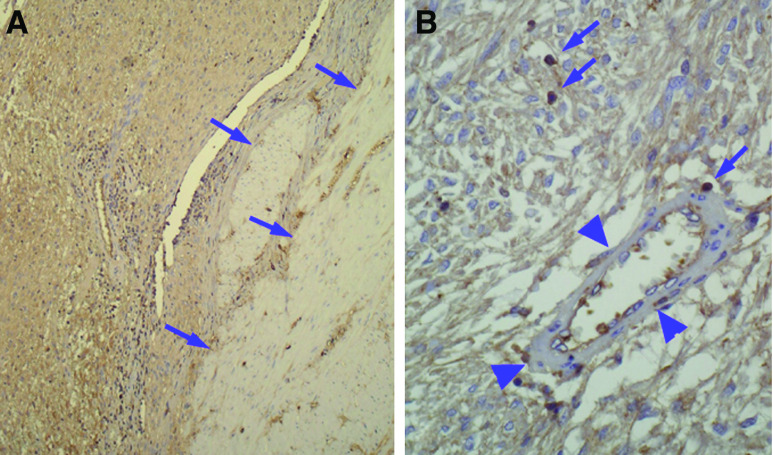
Detection of SCF in GISTs and nontumour cells. Immunohistochemistry was performed on large GIST samples with anti-SCF. (**A**) The intensity of staining of the muscularis propria (arrows) was lower than that of GIST. (**B**) Within GISTs, some lymphocytes had a strong staining (arrows), while arterial walls were almost negative (arrow heads).

Immunohistochemistry performed on larger histological samples, which contained GIST as well as nontumour adjacent tissue, confirmed the diffuse staining within GIST and also showed a positivity in some nontumour cells. Staining of muscular cells of the muscularis propria and of the arteries was always lower than that of GIST cells (Figure 6). By contrast, some lymphocytes had a higher positivity (Figure 6).

## DISCUSSION

KIT plays a major role in GIST oncogenesis. Indeed, 95% of GISTs express KIT (Corless *et al*, 2004), and its inhibitor, Imatinib, shows strong antitumour effects (Demetri *et al*, 2002). About 70% of GISTs display gain-of-function mutation of *KIT* (Corless *et al*, 2004) responsible for constitutive KIT activation (Hirota *et al*, 1998). These mutations also play a major role in GIST oncogenesis. Indeed, they are associated with some familial cases (Nishida *et al*, 1998), and similarly *KIT*-mutated knock-in mice display high GIST incidence (Sommer *et al*, 2003). However, most data dealing with KIT activation by gain-of-function mutations are obtained in cells containing a homozygous mutation, while 94% of GISTs' mutations are heterozygous (Emile *et al*, 2004). It is, thus, not yet known whether heterozygous *KIT* mutations act by inducing overall KIT activation or by other mechanisms, such as activation of specific signal transduction pathways in GIST cells.

Our analysis of the activation status of KIT in 17 KIT-positive GISTs by the detection of phosphorylated tyrosine residues shows that at least a fraction of KIT was activated in each tumour, even in the absence of gain-of-function mutation, consistent with previously published data (Rubin *et al*, 2001; Heinrich *et al*, 2003b; Antonescu *et al*, 2005). After immunoprecipitation and adjustment of the total KIT amounts in each sample, we were able to quantify the activated fraction of KIT. In these conditions, we found no correlation with the mutational GIST's status. Thus, the heterozygous gain-of-function mutations of *KIT* in GISTs have a few impact on the overall activation of this tyrosine kinase receptor. These results prompted us to look for an alternative mechanism of KIT activation in GISTs.

The absence of correlation between mutational status and KIT activation could be explained by the presence of SCF within tumours. Indeed, we show that SCF transcripts were present in the entire GIST samples tested, results consistent with a recently published *c*DNA array study (Antonescu *et al*, 2004). We also detected SCF by ELISA and immunohistochemistry in up to 93% of GIST samples. SCF treatment of GIST544 cells, which express a heterozygous *KIT* exon 9 mutation induces a stronger KIT tyrosine phosphorylation (Duensing *et al*, 2004), while SCF treatment of GIST882 cells, which carry a homozygous *KIT* exon 13 mutation does not (Lux *et al*, 2000). The two main isoforms of *KIT*, GNNK− and GNNK+, have been shown to have differential biological activities. Indeed GNNK−, which is the most abundant in GISTs (Théou *et al*, 2004), strongly promotes colony formation in semisolid medium, loss of cell to cell contact inhibition, and tumour growth in nude mice (Caruana *et al*, 1999). Furthermore, after ligand stimulation, the GNNK− isoform displayed more rapid and extensive tyrosine autophosphorylation and faster internalization (Caruana *et al*, 1999). Thus SCF may activate the wild-type species of KIT in GISTs without mutations, and may also modulate the fraction of activated KIT in GISTs with heterozygous mutations.

The presence of both KIT and SCF has already been reported in several tumours (Hibi *et al*, 1991; Pietsch, 1993; Inoue *et al*, 1994; Hines *et al*, 1995; Kondoh *et al*, 1995; Lahm *et al*, 1995; Krystal *et al*, 1996; Ricotti *et al*, 1998; Zheng *et al*, 2004), suggesting either an autocrine or paracrine oncogenic effect. The cellular origin of SCF in GIST samples appears to be the tumour cells themselves. Immunohistochemistry disclosed a strong staining of most GIST cells with anti-SCF antibody. This staining, however, could result from the internalization of SCF after its ligation to KIT. Indeed, SCF is known to be produced by different cell types, and we did detect SCF mRNA in all the nontumoural digestive samples tested. This result highlights that microenvironnement of tumour cells could led to a paracrine loop mechanism. However, by establishing primary homogenous GIST cell cultures, we confirmed the SCF production, both membrane and soluble isoforms of SCF mRNA, in GIST cells.

In conclusion, although *KIT* gain-of-function mutations play a major role in GIST oncogenesis, we show that KIT activation in these tumours is unrelated to the presence of these mutations, and may result from an autocrine/paracrine mechanism. Therefore, heterozygous *KIT* mutations in GISTs may act by inducing specific signal transduction pathways, rather than enhancing overall KIT activation.

## Figures and Tables

**Table 1:** *KIT* mutations and KIT expression and activation in GISTs

**Patients**	**Primary site**	**Mutations**	**KIT^a^**	**phosphoKIT^a^**
70810	Stomach	KIT: del555-558	208 043	657 690
71100	Stomach	KIT: del553-558	400 505	152 088
71101	Stomach	KIT: del576	198 769	143 520
71175	Stomach	KIT: W557R	362 286	46 980
71181	Intestine	KIT: ins502-503	317 449	229 884
71229	Stomach	KIT: del576	482 715	36 444
71231	Intestine	KIT: del557-558	158 166	341 288
71237	Stomach	KIT: del561-578	87 749	257 960
71562	Stomach	KIT: del557-558	213 413	156 411
71224	Stomach	PDGFRA: del842-845	32 597	679 888
71103	Colon	wt	567 685	65 588
71105	Peritoneum	wt	363 408	55 359
71183	Stomach	wt	58 616	269 632
71186	Intestine	wt	36 972	1 046 213
71233	Colon	wt	7339	979 572
71385	Intestine	wt	197 507	152 647

a KIT and phosphoKIT correspond to the chemiluminescent intensity mesured on blots and repported to 18 *μ*g of total protein extract for KIT, and a chemoluminescent signal of total KIT at 7000 for phosphoKIT.
